# Dupilumab-induced pityriasis rosea in a 28-year-old male with atopic dermatitis

**DOI:** 10.1016/j.jdcr.2023.12.019

**Published:** 2024-01-19

**Authors:** Faraz Yousefian, Margaret Hurley, Liaqat Ali, Marcus Goodman, Katherine Rupley

**Affiliations:** a Goodman Dermatology, Roswell, Georgia; b Department of Dermatology Residency, Philadelphia College of Osteopathic Medicine, Philadelphia, Pennsylvania; c Pinkus Dermatopathology Laboratory, Monroe, Michigan

**Keywords:** atopic dermatitis, dupilumab, pityriasis rosea

*To the Editor:* We read with great interest the report by Zabel et al^1^ on dupilumab-induced pityriasis rosea (PR). We aim to expand on this finding with our case of dupilumab-induced PR in a 28-year-old male with atopic dermatitis (AD).

A 28-year-old male with a past medical history of asthma and AD presented with diffuse, frequent pruritic flares that did not fully respond to topical steroids, tacrolimus, and ruxolitinib. Given his partial improvement, he was started on dupilumab, the United States Food and Drug Administration-approved medication for AD, with 600 mg subcutaneous injection and 300 mg every 2 weeks for maintenance. At his 2-week follow-up, the patient endorsed having a fever and headache, which was followed by the development of enlarging, diffuse itchy rash that was different than his previous AD flares and that did not respond to oral antihistamines. The patient denied any shortness of breath, recent travel, sick contacts, and medication changes.

Physical examination revealed diffuse erythematous papules coalescing into plaques scattered throughout the body (Fig 1). A punch biopsy was performed, which revealed a subacute spongiotic dermatitis with lymphocytic exocytosis and the periodic acid–Schiff stains were negative for fungi, consistent with differential diagnosis of PR and AD (Fig 2). The patient was advised to hold the dupilumab and resume the previous topical treatments.

**Fig 1:**
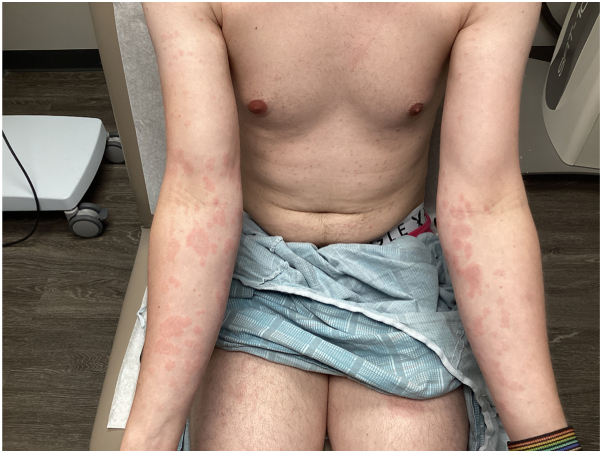
Physical examination revealing erythematous papules and coalescing plaques on upper and lower extremities post 2-week post dupilumab 600 mg subcutaneous loading dose injection.

**Fig 2:**
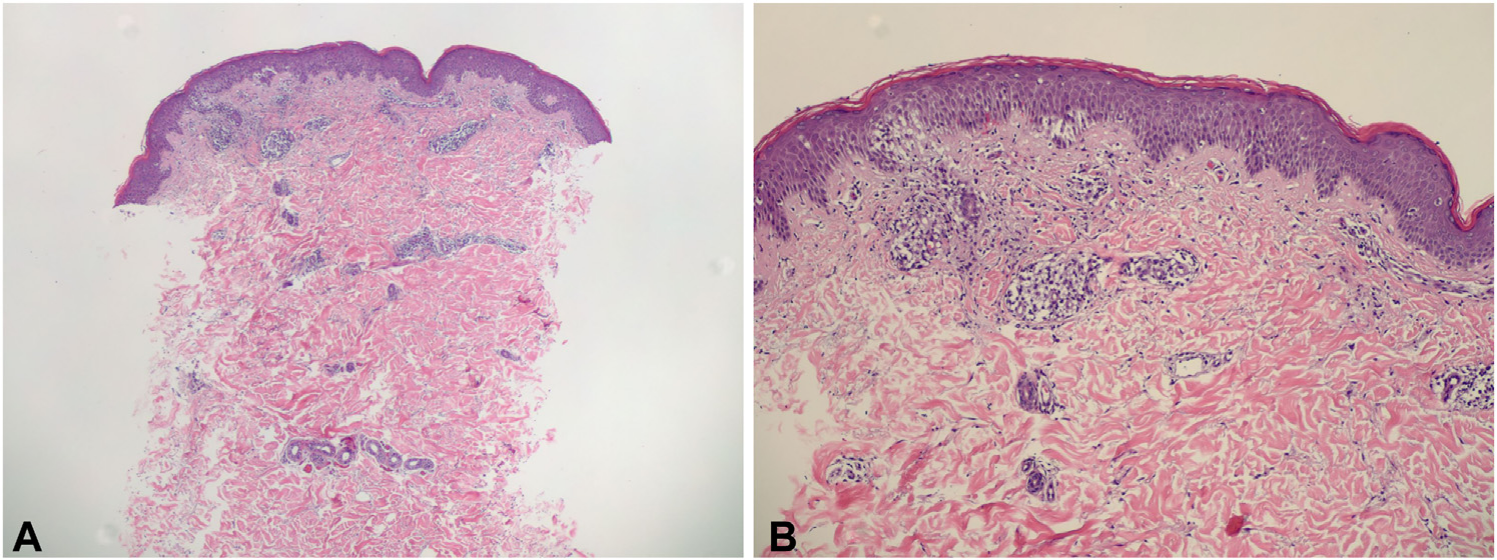
Histology showing subacute spongiotic dermatitis with lymphocytic exocytosis consistent with differential diagnosis of pityriasis rosea and atopic dermatitis. Mild epidermal hyperplasia with small foci of spongiosis, occasional formation of microscopic vesicles, overlying the areas of spongiosis are mounds of parakeratosis, and dermal perivascular lymphocytic inflammatory cell infiltrate with extravasated erythrocytes are seen in low (×10) and high power (×40) in hematoxylin and eosin stain.

At the 2-week follow-up visit, the patient wanted to proceed with dupilumab treatment because of the improvement of the recent rash and reemergent of his eczema. The patient resumed dupilumab at the maintenance dose. However, the same PR-like rash recurred at his following visit. Dupilumab was stopped again, and his PR-like rash resolved while his AD patches remained at this follow-up. Although he had symptoms of headache and fever, our patient’s presentation with coalesced plaques on nonclassical regions of PR including the extremities and complete resolution of the rash following discontinuation of dupilumab supported the diagnosis of dupilumab-induced PR.

The safety and efficacy of dupilumab for AD has shown promise for extended use, up to 76 weeks.^2^ Frequently reported adverse events include injection site reactions, conjunctivitis, and other infections including upper respiratory tract infections and nasopharyngitis.^3^ It is important to differentiate classic PR versus PR-like drug reaction, as classic PR can still develop independent of drug therapy. PR-like drug reaction is described as more pruritic with confluent lesions that appear frequently on nonclassical regions, including the extremities and face, which resolve within 2 weeks of discontinuation of the causative drug.^4^

To our knowledge, there has only been one other report of a PR-like eruption attributed to dupilumab.^1^ Other medications that have been reported to induce a PR rash include barbiturates, captopril, isotretinoin, non-steroidal anti-inflammatory drugs, tyrosine kinase inhibitors, and several biologics including rituximab, imatinib, and adalimumab.^1^^,^^5^ Notably, the literature does not describe a PR-like eruption from another United States Food and Drug Administration-approved monoclonal antibody for AD, tralokinumab. For cases of PR-like drug reaction it is generally advised to discontinue the offending medication, while in cases of classic PR occurring independent of drug therapy the medication can be continued and monitored closely.^1^

## Conflicts of interest

None disclosed.
